# GEARBOCS: An Adeno Associated Virus Tool for *In Vivo* Gene Editing in Astrocytes

**DOI:** 10.1101/2023.01.17.524433

**Published:** 2023-01-19

**Authors:** Dhanesh Sivadasan Bindu, Christabel Xin Tan, Justin T. Savage, Cagla Eroglu

**Affiliations:** 1 Department of Cell Biology, Duke University Medical Center, Durham, NC 27710, USA.; 2 Department of Neurobiology, Duke University Medical Center, Durham, NC 27710, USA.; 3 Duke Institute for Brain Sciences (DIBS), Durham, NC 27710, USA.; 4 Howard Hughes Medical Institute, Duke University, Durham, NC, 27710, USA.

**Keywords:** CRISPR, Astrocytes, Cortex, Synapse, Vesicles, Exocytosis

## Abstract

In the mammalian central nervous system (CNS), astrocytes are indispensable for brain development, function, and health. However, non-invasive tools to study astrocyte biology and function *in vivo* have been limited to genetically modified mice. CRISPR/Cas9-based genome engineering enables rapid and precise gene manipulations in the CNS. Here, we developed a non-invasive astrocyte-specific method utilizing a single AAV vector, GEARBOCS (Gene Editing in AstRocytes Based On CRISPR/Cas9 System). We verified GEARBOCS’ specificity to mouse cortical astrocytes and demonstrated its utility for three types of gene manipulations: knockout (KO); tagging (TagIN); and reporter gene knock-in (Gene-TRAP) strategies. We deployed GEARBOCS to determine whether cortical astrocytes express Vamp2 protein. The presence of Vamp2-positive vesicles in cultured astrocytes is well-established, however, Vamp2 protein expression in astrocytes *in vivo* has proven difficult to ascertain due to its overwhelming abundance in neurons. Using GEARBOCS, we delineated the *in vivo* astrocytic Vamp2 expression and found that it is required for maintaining excitatory and inhibitory synapse numbers in the visual cortex. GEARBOCS strategy provides fast and efficient means to study astrocyte biology *in vivo*.

## Graphical Abstract

- GEARBOCS is a single AAV-based CRISPR tool to target mouse astrocytes *in vivo*.
- GEARBOCS can be used to knockout, tag, or gene-trap genes of interest in astrocytes *in vivo*.
- Using GEARBOCS, we confirmed astrocytes express Vamp2 and found that astrocytic Vamp2 is required for maintenance of excitatory and inhibitory synapse numbers *in vivo*.

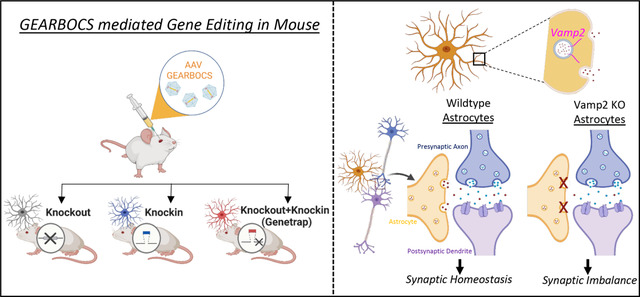

## Introduction

CRISPR-Cas9 (i.e., Clustered regularly interspaced short palindromic repeats-associated endonuclease 9) mediated gene-editing is widely used to engineer the mammalian genome (Doudna and Charpentier, 2014; Guan et al., 2022; Hsu et al., 2014; Knott and Doudna, 2018). Cas9 cleaves the genome at specific guide RNA (gRNA) target sites generating double-strand breaks. These breaks are repaired by two cell-intrinsic repair mechanisms: non-homologous end-joining (NHEJ) or homology-directed repair (HDR) (Heyer et al., 2010; Mao et al., 2008). Cas9-mediated cleavage events continue until repair induces insertions or deletions in the genome (i.e., indels) precluding gRNA recognition and further cleavage by Cas9. Indels within coding sequences often result in frameshifts and premature stop codons generating gene knockouts. HDR and NHEJ following CRISPR-Cas9-mediated genome cleavage can also be used to introduce engineered sequences into the genome even in postmitotic cells such as neurons (Suzuki and Izpisua Belmonte, 2018; Suzuki et al., 2019). These knock-in strategies utilize in-frame donor sequences to insert reporters or tags within the open reading frames of target genes.

Numerous methods, such as SLENDR (Mikuni et al., 2016), vSLENDR (Nishiyama et al., 2017), HITI (Suzuki et al., 2016), HiUGE (Gao et al., 2019), and ORANGE (Willems et al., 2020) were developed for gene knockout and knock-in applications in neurons, each using different DNA delivery methods or molecular strategies for donor insertion. For example, HDR-based SLENDR and vSLENDR use *in utero* electroporation or Adeno Associated Virus (AAV)-based methods to deliver gene-editing machinery into the mitotic radial glial stem cells or postmitotic neurons (Mikuni *et al.*, 2016; Nishiyama *et al.*, 2017). However, the efficiency of HDR-mediated donor insertion in postmitotic cells is limited (Mikuni *et al.*, 2016). The homology-independent targeted integration (HITI) method offers more effective genome editing and endogenous tagging of proteins of interest in postmitotic cells such as neurons (Suzuki *et al.*, 2016). Homology-independent Universal Genome Engineering (HiUGE) utilizes universal guide/donor pairs to enable rapid alterations of endogenous proteins without the need for designing target-specific donors (Gao *et al.*, 2019). Even though these gene-editing tools have versatile *in vitro* and *in vivo* applications, they depend on multiple vectors harboring the essential components required for genome engineering. Moreover, these strategies are developed primarily for neuronal gene manipulation.

In the CNS, non-neuronal glial cells, called astrocytes, play critical roles in neuronal connectivity and health. Astrocytes are characterized by their complex, ramified structures which are arborized into several thin filamentous branches (Halassa et al., 2007; Oberheim et al., 2006). Astrocytes are indispensable for the functioning of the brain. They provide trophic support to neurons, maintain the blood-brain barrier, regulate ion/water homeostasis as well as control synaptic transmission (Heithoff et al., 2021; Lyon and Allen, 2021; Mederos et al., 2018; Olsen et al., 2015). These essential roles of astrocytes in synapse formation, maturation, elimination, and maintenance depend on a complex bidirectional interaction with neurons (Chung et al., 2015; Shan et al., 2021) mediated by secreted and adhesion molecules (Saint-Martin and Goda, 2022; Tan and Eroglu, 2021).

Despite the undeniable importance of astrocytes in brain development and function, our molecular understanding of this glial cell type is still in its infancy. To address this knowledge gap, many recent studies revealed astrocyte-specific gene expression profiles and proteomes in the mammalian brain both in normal and disease conditions (Batiuk et al., 2020; Bayraktar et al., 2020; Endo et al., 2022; Farhy-Tselnicker et al., 2021; Rayaprolu et al., 2022; Takano et al., 2020; Zhang et al., 2014; Zhang et al., 2016; Chai et al., 2017; Endo *et al.*, 2022). These -omics approaches provide a rich resource for future discoveries; however, efficient tools for rapid *in vivo* genome editing of mouse astrocytes are needed to speed up discovery.

Here, we developed an astrocyte-specific CRISPR-based gene-editing tool, GEARBOCS (Gene Editing in AstRocytes Based On CRISPR/HITI System) to address this need. GEARBOCS is a single AAV vector to be used in conjunction with transgenic mice expressing Cas9 in a Cre-dependent manner. GEARBOCS enables three genome engineering strategies: Gene Knockout (KO), knocking in a tag for labeling of endogenous proteins (TagIN) and, simultaneous KO and reporter knock-in (GeneTRAP). Importantly, GEARBOCS harbors all essential components for genome editing in a single AAV backbone. We tested the efficiency and specificity of GEARBOCS for *in vivo* gene editing and applied it to test whether astrocytes express Vesicular associated membrane protein 2, Vamp2 *in vivo*.

Vamp2 (also known as synaptobrevin II) is a component of the soluble NSF attachment protein receptor (SNARE) complex and its functions at neuronal synapses for regulation of neurotransmitter release are well-characterized (Salpietro et al., 2019; Wang et al., 2020; Zhang *et al.*, 2014). However, the expression and functional relevance of Vamp2 and the role of the SNARE complex in astrocytes has been controversial (Slezak et al., 2012; Yamamoto et al., 2012). Using GEARBOCS, we verified the expression of Vamp2 in astrocytes *in vivo* and tested its roles in controlling synapse numbers in the mouse cortex.

## Results

### GEARBOCS is designed as a single AAV tool for *in vivo* genome editing in astrocytes.

**Figure 1:**
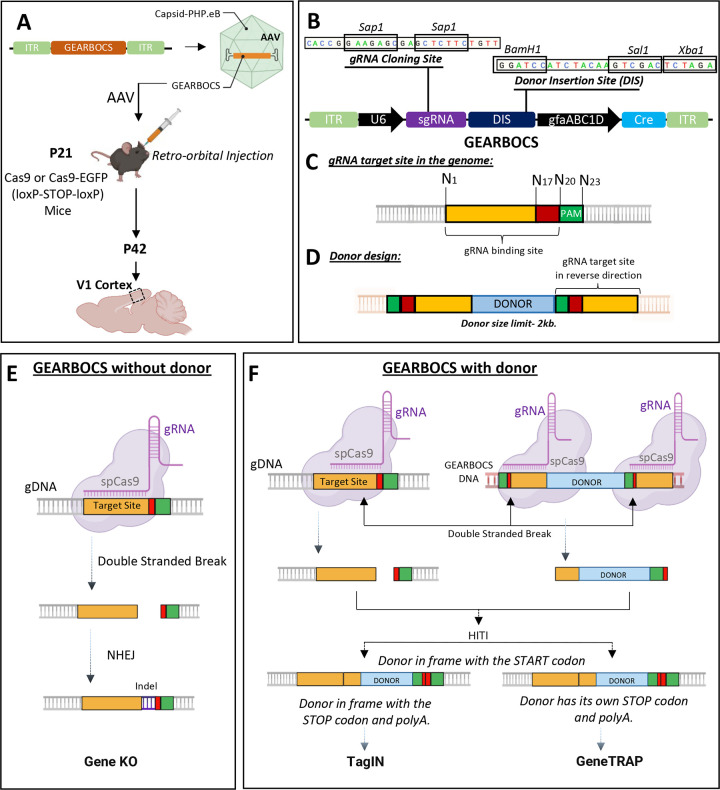
Development of GEARBOCS and its Applications: **A)** Experimental scheme for GEARBOCS-mediated *in vivo* genome editing in cortical astrocytes. GEARBOCS AAVs are produced with AAV-PHP.eB capsid and retro-orbitally injected into the loxP-STOP-loxP Cas9 or Cas9-EGFP mice at P21. Brains are prepared for immunohistochemistry and subsequent confocal fluorescent imaging at P42. **B)** Schematic of GEARBOCS vector showing its four essential elements for CRISPR/Cas9-based genome editing: 1) A human U6 promoter that can drive the expression of a unique guide RNA (gRNA) which is cloned into the gRNA cloning site (*Sap1* restriction enzyme); 2) Donor Insertion Site (DIS) wherein the donor DNA (e.g. encoding epitope tags, EGFP, mCherry etc.) can be cloned between the BamH1 and Sal1 restriction sites, 3) Cre expression cassette driven by astrocyte-specific gfaABC1D promoter; 4) AAV2 Inverted terminal repeats (ITR) for AAV packaging and expression. **C).** Guide RNA target site present in the genome is recognized by the unique gRNA/Cas9 complex through its recognition sequence and PAM sequence (green box) followed by the double stranded break at cut site (between red and yellow box). **D).** Donor DNA designed to clone into the GEARBOCS has the donor sequence (Light blue Box) flanked by the guide RNA target sites at both ends. GEARBOCS can accommodate up to 2kb size donors for AAV mediated cargo delivery and *in vivo* genome editing. **E-F** Schematic of GEARBOCS mediated genome editing mechanism in astrocytes. In GEARBOCS without donor model, AAV mediated delivery of GEARBOCS into the mouse cortex in loxP-STOP-loxP Cas9 mice leads to the Cre-mediated expression of spCas9 in astrocytes through the gfaABC1D promoter in GEARBOCS. **E**) In the absence of a donor sequence, U6 driven gRNA, and spCas9 cause double strand breaks in the gene of interest, which is followed by the imprecise non-homologous end joining (NHEJ) repair process, leading to indels and subsequent gene knockout (KO). **F)** In GEARBOCS with donor, guided by the gRNA, spCas9 makes double stranded breaks both within the gene of interest and the GEARBOCS vector around the donor sites. The excised donor fragment from the GEARBOCS can integrate into the genome by homology-independent targeted integration (HITI). The donors are designed to be in frame with the gene of interest. If the endogenous tagging of a protein of interest in astrocytes (TagIN) is needed, the tag is knocked in frame with both the START and STOP codons of the gene of interest. If the donor has its own STOP codon and polyA tail, then this will lead to the GeneTRAP.

To facilitate rapid, astrocyte-specific, *in vivo* genome editing, we designed a CRISPR tool within a single AAV vector, which we named GEARBOCS. GEARBOCS vector is designed to be used in combination with an AAV capsid such as PHP.eB (Chan et al., 2017) which is delivered into the CNS of mice via retro-orbital injection. For GEARBOCS strategy to work, transgenic mice that express spCas9 in a Cre recombinase-dependent manner are required (Figure 1A). For all the experiments, we used GEARBOCS AAVs produced with AAV-PHP.eB capsids and retro-orbitally injected them into the Cas9 (loxP-STOP-loxP) or Cas9-EGFP (loxP-STOP-loxP) mice at postnatal day (P) 21 (Figure 1A). After 3 weeks at P42, brains were harvested and analyzed. For consistency, we focused our analyses to the V1 visual cortex.

The GEARBOCS vector (5743bp) harbors four essential components for *in vivo* genome editing (Figure 1B)- 1) A U6 expression cassette, including a human U6 promoter, a gRNA cloning site, and a 76bp gRNA scaffold (Ran et al., 2013). U6 promoter drives the expression of a small guide RNA (gRNA), which is cloned into the *Sap1* restriction site within the gRNA cloning site (Figure 1B). The gRNA is designed to match a 20bp genomic target site, which is next to a Protospacer Adjacent Motif (PAM) (Figure 1C). 2) A Donor Insertion Site (DIS) wherein the donor DNA can be cloned between the BamH1 and Sal1 restriction sites (Figure 1B). The donor DNA is omitted for generating gene KOs. Alternatively, donor sequences, ranging from a small epitope tag, like HA or V5, or a larger reporter tag, such as EGFP or mCherry (up to 2kb), can be cloned into the DIS. The donor sequence is flanked with the guide RNA target site sequences on both ends (Figure 1D). 3) A synthetic human glial fibrillary acidic protein (GFAP) promoter (Lee et al., 2008), gfaABC1D, to drive the expression of Cre in astrocytes. Cre removes the STOP codon and turns on Cas9 or Cas9-EGFP expression in astrocytes. 4) Two inverted terminal repeats (ITRs) for AAV packaging. The ITR sequences in GEARBOCS are based on the AAV2 serotype (Rabinowitz et al., 2002).

The GEARBOCS approach uses the intrinsic DNA repair mechanism, NHEJ. Thus, when combined with HITI of donor sequences (Suzuki *et al.*, 2016), GEARBOCS permits gene knock-in both in mitotic and postmitotic cells. GEARBOCS tool can be used to manipulate the astrocyte genome in three different ways. GEARBOCS, without a donor (Figure 1E), generates knock out (KO) of the gene of interest in astrocytes. The gRNA specific to the gene of interest guides the spCas9 in astrocytes, to the genomic locus to create a double stranded break upstream of its PAM sequence (i.e., NGG). This double stranded break is repaired by NHEJ, until the repair causes indels which results in a frame shift and gene KO. To generate KOs, we choose unique gRNAs targeting genomic regions close to the start codon of the gene of interest. gRNAs are chosen based on their low off-target and high on-target scores predicted by the Broad Institute CRISPick tool (https://portals.broadinstitute.org/gppx/crispick/public).

GEARBOCS can also be used for knocking in a donor sequence in frame with the gene of interest (Figure 1F). To do so, a donor sequence, flanked with the reverse-oriented genomic gRNA target sites on both ends, is cloned into the DIS of the GEARBOCS vector (Figure 1B and D). The gRNAs guide spCas9s to the genomic loci and the donor sequence within the viral genome to generate double-stranded breaks in both sequences. These cleavage events result in two blunt ends in the genome and two at the donor fragment (Figure 1F). The donor DNA is then integrated into the genomic target site via HITI (Suzuki *et al.*, 2016).

GEARBOCS with donor can be used for endogenous tagging of proteins of interest (TagIN) in astrocytes. To do so, the donor is designed to insert a protein coding sequence in frame with the endogenous coding sequence so that the endogenous start and stop codons and the polyA tails are kept intact (Figure 1F).

When the donor sequence is designed to insert in frame following the endogenous START site but has its own STOP codon and a synthetic polyA tail, this will lead to a gene trap with a subsequent knockout (Figure 1F). This GeneTRAP approach is particularly useful to label and visualize astrocytes in which a gene of interest is knocked out. Reverse orientation of gRNA target sequences flanking the donor (Figure 1D) is critical for avoiding further cleavage, thereby facilitating the insertion of donor in correct orientation (Suzuki and Belmonte, 2018). The GEARBOCS vector design enables us to interchange the donors quickly with a single cloning step. Hence, it provides an “all-in-one” simple and powerful CRISPR tool to target astrocytes *in vivo*.

### GEARBOCS enables efficient astrocyte-specific genome editing.

GEARBOCS uses the astrocyte-specific minimal promoter, gfaABC1D (Lee *et al.*, 2008) to drive the Cre expression and turn on the production of Cas9 or Cas9-EGFP in astrocytes of the commercially available Cas9 mice (Chiou et al., 2015; Platt et al., 2014). This approach overcomes the difficulty of expressing large-sized Cas9 orthologs through the AAV system.

**Figure 2:**
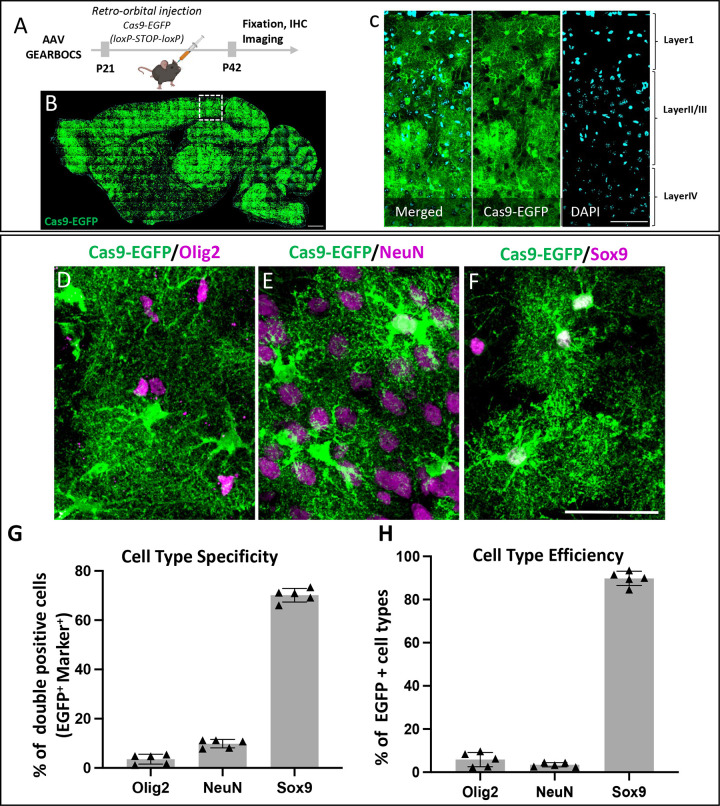
GEARBOCS efficiently targets mouse cortical astrocytes: **A).** Schematic of the experimental scheme. GEARBOCS AAV is generated with AAV-PHP.eB capsid and retro-orbitally injected into the loxP-STOP-loxP Cas9-EGFP mice at P21. Three weeks post viral injection (P42), brains are collected for analysis. **B)** Confocal immunofluorescent image of a sagittal section from AAV-GEARBOCS-injected loxP-STOP-loxP Cas9-EGFP mouse brain showing the prevalence of Cas9-EGFP expression (green). Scale bar=50μm. **C).** Confocal immunofluorescent image from V1 cortex (indicated as dotted box in **B**) showing the Cas9-EGFP positive cells in different cortical layers. Scale bar=30μm. **D-F)** Confocal immunofluorescent images showing EGFP positive cells (green) co-stained with different cell type-specific markers (magenta) such as **(D)** Olig2 (oligodendrocytes), **(E)** NeuN (Neurons) and **(F)** Sox9 (astrocytes). Scale bar=20μm. **G)** Cell type-specificity of GEARBOCS-Cre expression quantified by as the percentage of each cell types co-localized with EGFP positive cells in the V1 cortex**. H)** Cell type efficiency of GEARBOCS-Cre expression quantified by taking the percentage of EGFP positive cells co-localized with cell type marker in the V1 cortex. (G-H: n=5 animals, Error bars show SEM).

Previous studies have raised the caveat that human gfaABC1D promoter may permit Cre-mediated genome recombination in some neuronal cells, when AAVs are directly injected at high titers into the mouse brain (O’Carroll et al., 2020). Thus, we first tested the astrocyte-specificity of GEARBOCS in the mouse cortex for its *in vivo* applications. To do so, we used the AAV capsid PHP.eB to package the GEARBOCS vector. This capsid efficiently transduces CNS cells (Chan *et al.*, 2017) when retro-orbitally injected, eliminating the need for invasive surgery and direct injection into the CNS. We found that retro-orbitally injected AAV-GEARBOCS indeed transduces the mouse brain with high efficiency which is evidenced by the abundance of Cas9-EGFP positive cells across many brain regions (Figure 2A–B) including V1 cortex (Figure 2C)

To investigate the astrocyte specificity of Cre-mediated Cas9 expression, we performed the co-immunostaining of Cas9-EGFP with different CNS cell type-specific markers. We used NeuN to label neurons (Gusel’nikova and Korzhevskiy, 2015), Olig2 for oligodendrocyte lineage cells (Yokoo et al., 2004) and Sox9 that labels the majority of the astrocytes (Sun et al., 2017) (Figure 2D–F). The quantitation of marker colocalization with the EGFP/Cas9-expressing (i.e., EGFP/Cas9+) cells in the cortex revealed that they were overwhelmingly Sox9+ (70.14±2.59%) compared to NeuN+ (9.83±1.60%) or Olig2+ cells (3.58±1.88%) (Figure 2G). Of all the Sox9+ astrocytes, 89.80±3.29% were EGFP/Cas9+, whereas only 3.47±0.98% and 5.86±3.31% percent of NeuN+ or Olig2+ cells were EGFP/Cas9+, respectively (Figure 2H). These results show that GEARBOCS targets Cas9 expression to cortical mouse astrocytes with considerable specificity and high efficiency.

### GEARBOCS-mediated gene editing of *Gfap* in mouse cortical astrocytes

**Figure 3:**
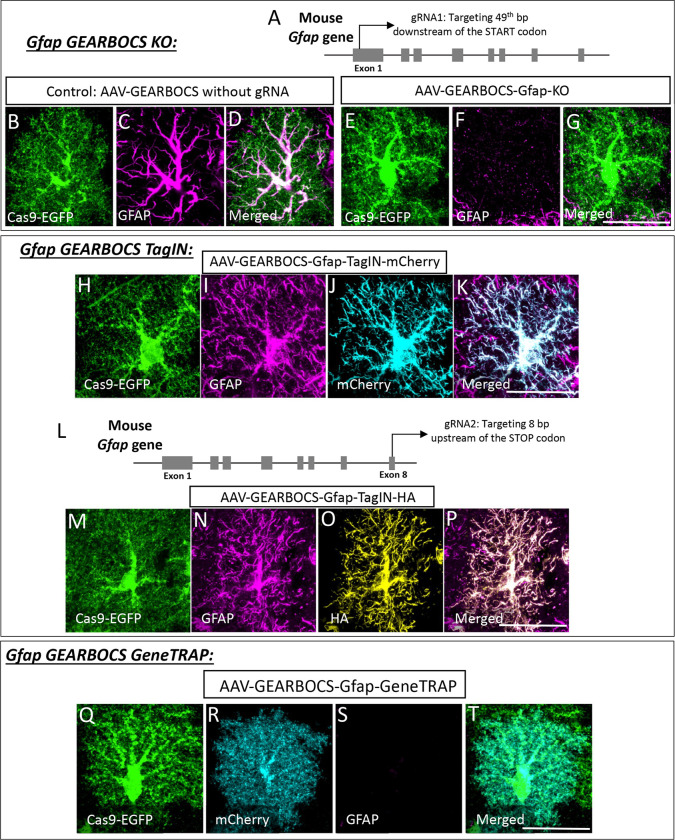
GEARBOCS-mediated editing of *Gfap* gene in astrocytes: **A).** Schematic showing the first gRNA target site in *Gfap* locus (gRNA1) used in this study. **B-D).** Confocal immunofluorescence microscopy images showing a Cas9-EGFP positive astrocyte (green) transduced with AAV-GEARBOCS vector without the gRNA (Control). This astrocyte still has GFAP staining (magenta). **E-G).** Confocal immunofluorescence microscopy images showing the loss of GFAP staining (magenta) in a Cas9-EGFP (green) positive AAV-GEARBOCS-*Gfap*-KO transduced astrocyte. **H-K)** Confocal immunofluorescence microscopy images of a Cas9-EGFP-positive astrocyte (green) transduced with the AAV-GEARBOCS-Gfap-TagIN-mCherry showing the co-localization of GFAP (magenta) with mCherry (cyan). **L)** Schematic showing the second gRNA (gRNA2) target site in the mouse *Gfap* gene. **M-P).** Confocal Immunofluorescence microscopy images of a Cas9-EGFP positive astrocyte (green) transduced with AAV-GEARBOCS-Gfap-TagIN-HA showing the co-localization of GFAP (magenta) with HA (gold). **Q-T).** Confocal immunofluorescent image of a Cas9-EGFP positive astrocyte (green) transduced with the AAV-GEARBOCS-Gfap-GeneTRAP the loss of GFAP staining in the mCherry (cyan) positive astrocyte. Scale bars=20μm.

To evaluate the effectiveness of GEARBOCS as a molecular CRISPR tool for manipulation of astrocyte genome in multiple ways, we first targeted *Gfap*. Gfap is a class-III cytoskeletal intermediate filament protein that is highly abundant in astrocytes (Yang and Wang, 2015). To knockout *Gfap* in astrocytes, we first applied the GEARBOCS KO strategy (Figure 1C). To do so, we selected a unique gRNA targeting the first exon, located 49bp after the start codon (Figure 3A). The selected gRNA1 was cloned into GEARBOCS to generate the AAV-GEARBOCS-Gfap-KO and it was retro-orbitally injected into P21 Cas9-EGFP (loxP-STOP-loxP) mice. We used AAV-GEARBOCS without a gRNA as our control. Immunostaining showed that the Gfap expression is ablated in EGFP/Cas9+ cells which are transduced by AAV-GEARBOCS-Gfap-KO when compared to the astrocytes transduced by the control virus (Figure 3B–G). This result shows that the GEARBOCS approach can be used to knockout gene(s) of interest in astrocytes.

To test the utility of GEARBOCS for TagIN applications (Figure 1D), we designed a donor sequence, containing an mCherry fluorophore tag sequence, flanked with two inverted gRNA target sequences on either side. This donor sequence was then cloned into the GEARBOCS vector, which already contained gRNA1, generating AAV-GEARBOCS-Gfap-TagIN-mCherry. AAVs were injected into the floxed-Cas9-EGFP mice and cortical sections were stained with antibodies against Gfap and mCherry. Indeed, the endogenous Gfap staining co-localized with mCherry in the GEARBOCS-transduced EGFP/Cas9+ astrocytes (Figure 3H–K).

With gRNA1, we targeted the N-terminal of Gfap protein for TagIN purposes; however, this approach can lead to the KO of the gene in some cells, if the donor integration does not happen at the locus prior to NHEJ. This caveat can be avoided by tagging proteins at their C-terminal. To demonstrate this possibility, we used another gRNA (gRNA2) (Gao *et al.*, 2019) to introduce a hemagglutinin (HA) epitope-tag at the C-terminal of the Gfap protein (Figure 3L). The donor sequence containing HA-tag and inverted gRNA target sequences were cloned into the GEARBOCS containing gRNA2 to generate AAV-GEARBOCS-Gfap-TagIN-HA. Upon AAV injection, we observed the co-localization of HA immunostaining with endogenous Gfap (Figure 3M–P). Altogether, these results demonstrate the usefulness of GEARBOCS to tag an astrocytic protein of interest, broadening its potential applications to study the astrocyte proteome.

Finally, we tested the GEARBOCS tool’s utility to achieve a GeneTRAP- i.e., the simultaneous KO of an astrocytic gene of interest and insertion of a reporter gene into its locus (Figure 1D). To do so, we used the gRNA1 targeting the *Gfap* gene in exon 1 (Figure 3E–G). We designed and generated the mCherry-CAAX reporter donor with its own STOP codon and polyA tail. This GeneTRAP strategy caused loss of endogenous Gfap expression, while the mCherry-CAAX was produced in these KO cells facilitating their visualization (Figure 3Q–T). Collectively, these results demonstrate that GEARBOCS is an efficient tool for gene KO, endogenous tagging, or gene trap purposes, greatly facilitating study of astrocyte cell biology in the mouse CNS. Hence, this “all-in-one” single AAV CRISPR tool could help address the limitations and challenges in understanding the development and function of astrocytes *in vivo*.

### Mouse cortical astrocytes express Vamp2 *in vivo*

**Figure 4:**
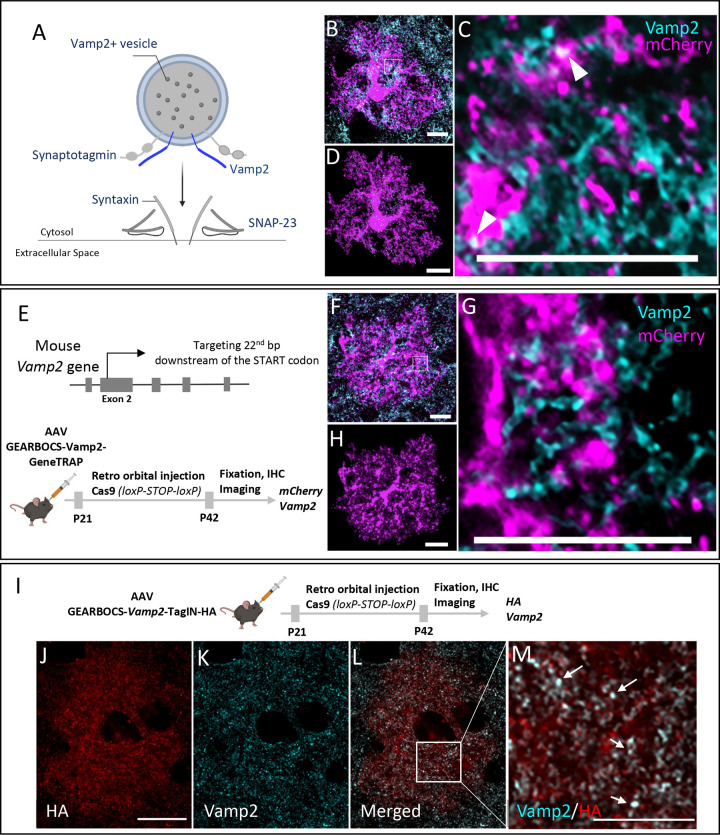
*In vivo* mouse cortical astrocytes express Vamp2: **A).** Schematic of Vamp2 (dark blue) as a member of the SNARE complex. **B)**. Confocal immunofluorescence microscopy image from an mCherry-positive astrocyte (magenta) transduced with AAV-gfaABC1D-mCherry-CAAX co-stained with a Vamp2 antibody (cyan). Scale bar=10μm. **C).** Zoom in image of a single optical section showing the presence of Vamp2 (cyan) inside the mCherry-CAAX filled astrocytes (magenta). Scale bar=10μm. **D).** IMARIS reconstructed image showing the spatial distribution of Vamp2 (cyan) within the mCherry-CAAX filled astrocyte domain (magenta); Scale bar-10μm. **E).** Schematic of mouse *Vamp2* gene showing the gRNA target site and the experimental scheme showing the GEARBOCS-*Vamp2*-GeneTRAP. AAV-GEARBOCS-*Vamp2*-GeneTRAP was retro-orbitally injected into the loxP-STOP-loxP Cas9 mice at P21 and immunohistochemical analyses of mCherry and Vamp2 were carried out at P42. **F).** Confocal immunofluorescence image of an mCherry positive astrocyte (magenta) from AAV-GEARBOCS-*Vamp2*-GeneTRAP transduced brain showing that Vamp2 staining within the mCherry-positive astrocyte is diminished. Scale bar=10μm. **G).** Zoom in image of a single optical section showing the reduction of Vamp2 staining inside the mCherry-CAAX positive GEARBOCS-*Vamp2*-GeneTRAP astrocyte (magenta). Scale bar-10μm. **H).** IMARIS reconstructed image showing the decreased expression of Vamp2 (cyan) in a GEARBOCS-*Vamp2*-GeneTRAP astrocyte (magenta). Scale bar-10μm. **I).** Schematic of the experimental scheme showing the GEARBOCS-*Vamp2*-TagIN with an HA tag. AAV-GEARBOCS-*Vamp2*-TagIN-HA was retro-orbitally injected into the loxP-STOP-loxP Cas9 mice at P21 and immunohistochemical analyses of HA and Vamp2 were carried out at P42. **J-L).** Confocal immunofluorescence image of a single optical section from an astrocyte transduced with the AAV-GEARBOCS-*Vamp2*-TagIN-HA showing the co-localization Vamp2 (cyan) with HA (Red). Scale bar-20μm. **M).** Zoom in image of a single optical section showing the co-localization of Vamp2 with HA (white). Scale bar=10μm.

Astrocytes secrete synapse-modulating proteins, peptides, and neuroactive small molecules (Verkhratsky et al., 2016; Allen and Eroglu, 2017; Baldwin and Eroglu, 2017); however, the mechanisms underlying their release are unknown. Vamp2 is an integral component of the SNARE complex which mediates calcium-dependent vesicular exocytosis (Salpietro *et al.*, 2019; Wang *et al.*, 2020). In response to an increase in cytosolic calcium levels, vesicular Vamp2 forms ternary SNARE complexes with the plasma membrane proteins such as syntaxin and SNAP23 to cause membrane fusion and release of vesicular cargo (Figure 4A).

While the presence of calcium transients in astrocytes have been well documented (Lia et al., 2021), the expression of Vamp2 in astrocytes and its functions are still controversial. This is primarily due to the overwhelming abundance of neuronal Vamp2 compared to astrocytes and the limited specificity of both chemical (McMahon et al., 1993; Yamamoto *et al.*, 2012) and genetic approaches to target astrocytic Vamp2 (Slezak *et al.*, 2012). Therefore, it has been difficult to discriminate the astrocytic expression of Vamp2 and its functions from its neuronal counterpart.

To capture astrocytic Vamp2 expression by conventional immunohistochemical methods and imaging, we immunostained Vamp2 in brain sections from mice in which cortical astrocytes were transduced with AAV-gfaABC1D-mCherry-CAAX. Co-localization of mCherry+ astrocytes with Vamp2 was detected; however, due to the intense Vamp2 staining in brain tissue and the resolution limit of light microscopy, it was difficult to confirm Vamp2 expression within astrocytes (Figure 4B–D). Using the Imaris software, we reconstructed the fluorescence signal from an mCherry-filled astrocyte to map the spatial Vamp2 expression in astrocytes (Figure 4D).

To verify the specificity of observed astrocytic Vamp2 staining with GEARBOCS, we used a Vamp2-targeting gRNA which was previously described (Horvath et al., 2017). Using the GeneTRAP method, we knocked-in an mCherry-CAAX donor with a STOP codon and polyA tail at its second exon, 22bp after the start codon (Figure 4E). This strategy allowed us to confirm the endogenous Vamp2 promoter activity driving mCherry-CAAX expression, while simultaneously knocking out Vamp2 expression. Immunohistochemical analysis of mCherry and Vamp2 in cortical astrocytes transduced with AAV-GEARBOCS-Vamp2-GeneTRAP revealed greatly diminished Vamp2 staining within the mCherry+ astrocytes (Figure 4F–H). These results show that Vamp2 locus is transcriptionally active in astrocytes further suggesting that astrocytes express Vamp2.

To further corroborate this finding, we applied GEARBOCS TagIN strategy to insert an HA epitope tag at the N-terminal of the Vamp2 protein. To do so, we used the same gRNA (Figure 4E), but changed our donor with an in-frame sequence for a single HA-epitope tag. AAV-GEARBOCS-Vamp2-TagIN-HA viruses were retro-orbitally injected into the Cas9 mice at P21 (Figure 4I) and brains were harvested at P42. HA-tagging revealed punctate staining within transduced astrocytes, suggesting a vesicular localization (Figure 4J). Coimmunostaining of astrocytes with HA and Vamp2 showed that Vamp2 signal colocalizes with the HA (Figure 4J–M). Altogether, these data show that cortical astrocytes do express Vamp2 *in vivo*.

### Astrocytic Vamp2 is required for maintenance of excitatory and inhibitory synapse numbers.

Astrocytes control the formation and maintenance of synapses through secreted and cell adhesion molecules (Allen and Eroglu, 2017; Saint-Martin and Goda, 2022). The synaptic functions of astrocytes are mediated through their intricate morphologies. In fact, astrocyte morphogenesis coincides with the period of accelerated synaptogenesis in the mouse visual cortex. Importantly, disruption of astrocyte-neuron cell adhesions not only impacts astrocyte morphology but also alters the fine balance between inhibition and excitation in the CNS (Freeman, 2010; Stogsdill et al., 2017). However, whether astrocytic vesicular exocytosis is involved in astrocyte morphology or synaptogenic function remains enigmatic. Because we found Vamp2 to be expressed in mouse cortical astrocytes (Figure 4), we investigated its role in cortical astrocyte morphology and synaptogenic ability.

**Figure 5:**
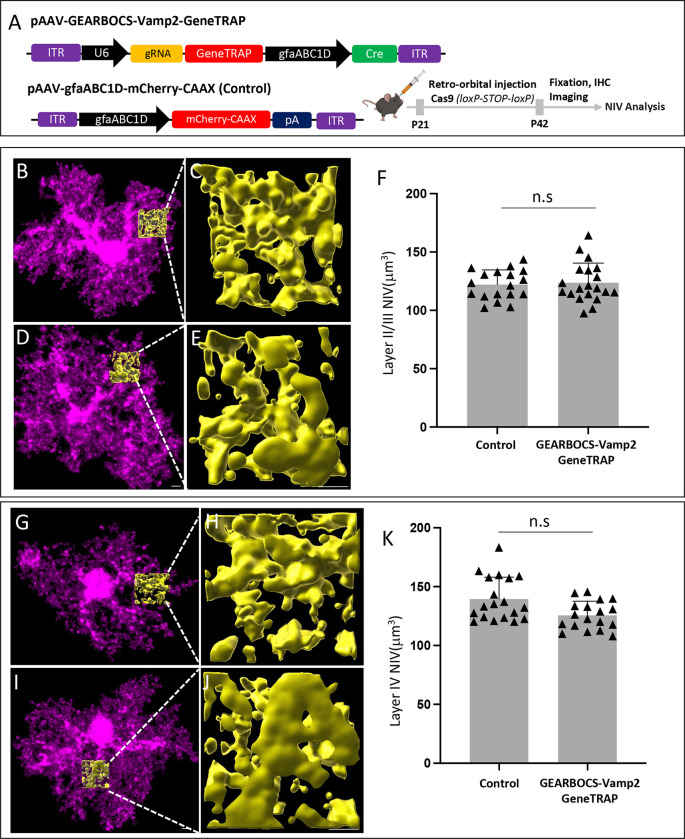
Astrocytic Vamp2 is not required for maintenance of astrocyte morphology: **A).** Schematic of both AAV-GEARBOCS-*Vamp2*-GeneTRAP and AAV-gfaABC1D-mCherry-CAAX (Control) plasmids and the experimental scheme of AAV injection and Neuropil Infiltration Volume (NIV) analyses. AAVs were generated with AAV-PHP.eB capsid and retro-orbitally injected into the loxP-STOP-loxP Cas9 mice at P21 and brains were collected at P42 for NIV analysis. Confocal immunofluorescence microscopy images of V1 Layer 2/3 **(B-E)** and Layer IV**(G-J)** mCherry positive WT and Vamp2-GeneTRAP astrocytes (magenta) and NIVs (gold). Quantification of average NIV from Layer II/III **(F)** and Layer IV **(K)** Control and *Vamp2*-GeneTRAP astrocytes. Nested t-test. Scale bar=1μm; 3 NIV/cell, 16–20 cells/condition, 5–6 mice/condition, Layer 2/3 P value- 0.7041, Layer IV P value- 0.0558.

To do so, we deployed the GEARBOCS-GeneTRAP method to KO astrocytic Vamp2 with concurrent mCherry-labeling of KO astrocytes. We retro-orbitally injected AAV-GEARBOCS-Vamp2-GeneTRAP (Figure 5A). To determine if astrocytic Vamp2 has any effects on astrocyte morphology, we analyzed how this genetic manipulation impacted neuropil infiltration by the fine perisynaptic astrocytic processes. These processes interact with synapses and their abundance per unit brain volume is a measure of astrocyte complexity (Baldwin et al., 2021; Stogsdill *et al.*, 2017; Takano *et al.*, 2020). We did not see any differences in the neuropil infiltration volume (NIV) from Vamp2 KO astrocytes compared to control in layer II/III (Figure 5B–F) and layer IV (Figure 5G–K). This result shows that astrocytic Vamp2 is not required to maintain astrocyte morphology. Next, we investigated if astrocytic Vamp2 is required to maintain proper synaptic connectivity. To do so, we quantified the numbers of excitatory and inhibitory synapses within the territories of Vamp2-GeneTRAP and control astrocytes. For synapse quantification, we used an immunohistochemical method that takes advantage of the close proximity of pre- and post-synaptic proteins at synaptic junctions (Baldwin *et al.*, 2021; Stogsdill *et al.*, 2017; Takano *et al.*, 2020). Even though these proteins are at different cellular compartments (i.e., axons and dendrites, respectively), they appear to co-localize at synapses due to the resolution limit of light microscopy.

**Figure 6:**
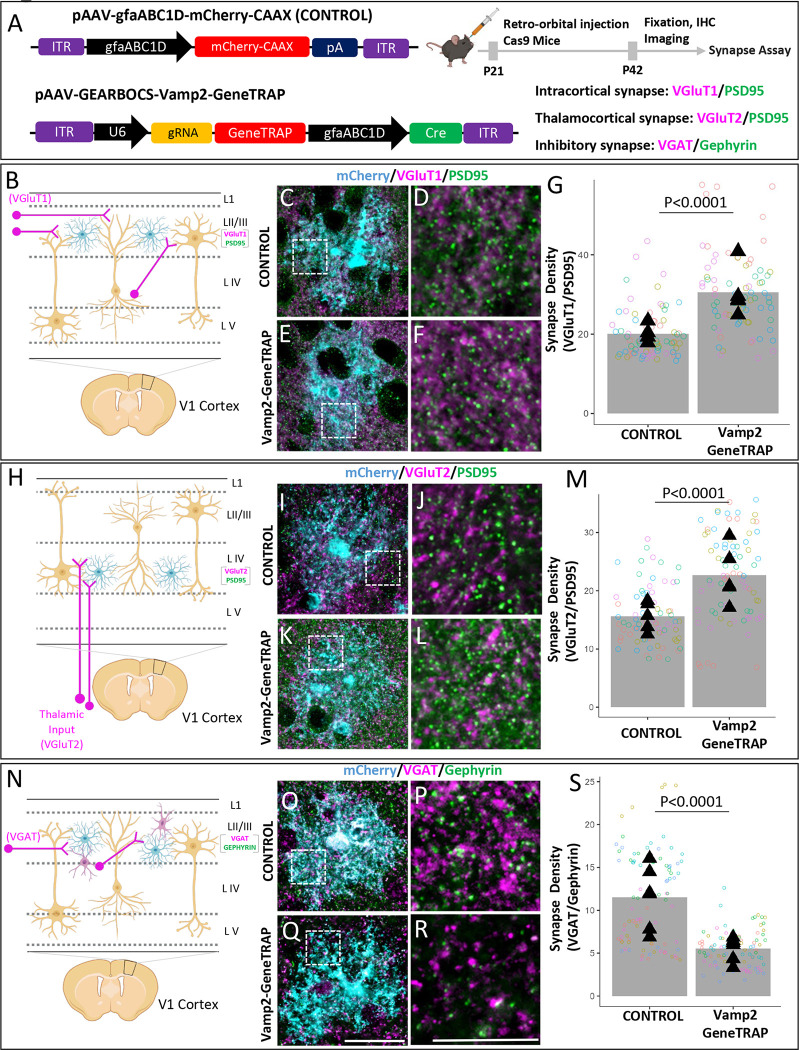
Astrocytic Vamp2 is required for regulating excitatory and inhibitory synapse numbers: **A).** Schematic of both AAV-GEARBOCS-*Vamp2*-GeneTRAP and AAV-gfaABC1D-mCherry-CAAX plasmid and the experimental scheme of AAV injection and synapse number quantification assays. AAVs were generated with the AAV-PHP.eB capsid and retro-orbitally injected into the loxP-STOP-loxP Cas9 mice at P21 and the brains were collected at P42. For synapse assays we used presynaptic (VGluT1 (intracortical), VGluT2 (thalamocortical) or VGAT) and postsynaptic (PSD95 or Gephyrin) markers specific to excitatory or inhibitory synapses. **B-S).** Schematic showing layer II/III VGluT1 (magenta) intracortical presynaptic inputs **(B)**, layer IV VGluT2 (magenta) thalamic presynaptic inputs **(H)** and layer II/III VGAT (magenta) inhibitory presynaptic inputs **(N)** in V1cortex. Confocal immunofluorescence images of intracortical excitatory synapses marked as close apposition of VGluT1 (magenta) and PSD95 (green); **C-F**), thalamocortical excitatory synapses (VGluT2 (magenta) and PSD95 (green); **I-L**) and inhibitory synapses (VGAT (magenta) and gephyrin (green); **O-R)** within the territory of mCherry positive Control or GEARBOCS-*Vamp2*-GeneTRAP astrocytes (cyan). Zoom-in image showing co-localized synaptic puncta (White) **(D, F, J, L, P, R).** Quantification of synaptic density of intracortical **(G)**, thalamocortical **(M)**, and inhibitory **(S)** co-localized puncta within the territories of mCherry positive WT and GEARBOCS-*Vamp2*-GeneTRAP astrocytes. 5 images/cell, 3 cells/mouse, 5–6 mice/genotype. Nested ANOVA. Data are means ± s.e.m. Scale bars=20μm (C, E, I, K,O,Q), and 10μm (D,F,J,L,P,R).

To quantify excitatory cortical synapses, we labeled the brain sections with excitatory presynaptic markers VGluT1 (intracortical) or VGluT2 (thalamocortical) (Kaneko and Fujiyama, 2002) and postsynaptic marker PSD95. The inhibitory synapses were identified as the co-localization of presynaptic VGAT and postsynaptic Gephyrin. Interestingly, loss of Vamp2 in astrocytes caused a significant increase in the densities of intracortical (VGluT1/PSD95+) (Figure 6B–G) and thalamocortical (VGluT2/PSD95+) (Figure 6H–M) excitatory synapses when compared to control astrocytes. On the contrary, loss of Vamp2 caused a severe reduction in the density of VGAT/Gephyrin+ inhibitory synaptic puncta within the territories of Vamp2 KO astrocytes in layer 2/3 (Figure 6N–S).

Taken together, our results elucidate a previously unknown role for Vamp2 in mouse cortical astrocytes in maintaining the balance between the excitatory and inhibitory synapses. These findings suggest that Vamp2 controls the release of synapse-modifying factors. Our findings also demonstrate that GEARBOCS is a useful genome-editing tool for investigation of astrocyte-mediated complex cellular and molecular mechanisms in CNS development and function.

## Discussion

To further our understanding of astrocyte biology in CNS homeostasis and function an efficient molecular tool for rapid *in vivo* genome-editing in mouse astrocytes is needed. In this study, we devised a HITI-based, single virus, CRISPR tool, GEARBOCS, to target mouse cortical astrocytes *in vivo* and demonstrated its multiple applications in manipulating the astrocyte genome.

GEARBOCS has several advantages and applications. First, GEARBOCS is a single AAV vector capable of carrying all the essential components required for *in vivo* genome editing when used in conjunction with a Cre-dependent Cas9 transgenic mouse line. Single vector GEARBOCS strategy ensures the simultaneous and efficient delivery of all components to the astrocytes. Second, GEARBOCS allows for multiple genome-editing strategies including, knocking out, or knocking in reporters or tags to determine astrocytic protein expression and localization for genes of interest. Furthermore, the GEARBOCS-mediated GeneTRAP strategy can be used to sparsely label and visualize the KO astrocytes to study their morphology and synapse interactions. Importantly, GEARBOCS can accommodate donor sizes up to 2kb. The donors can be interchanged in GEARBOCS to generate KO, TagIN and GeneTRAP options for a gene of interest quickly. Third, GEARBOCS, when used with AAV capsids such as PHP.eB, efficiently and broadly targets astrocytes across the CNS after non-invasive retro-orbital injections. The high efficacy of astrocytic transduction provides a quick cell-type specific gene manipulation capability. Noninvasive AAV delivery greatly reduces the risk of causing reactive astrocytosis, which is often observed following direct injection of viruses into the brain (Ortinski et al., 2010).

AAV-mediated GEARBOCS delivery provides efficient transduction of the mouse cortical astrocytes compared to other cell types in the mouse brain. However, we observed a small number of neurons to express Cre despite the presence of the astrocyte-specific gfaABC1D promoter. Cre-mediated recombination of floxed Cas9-EGFP can occur even in the presence of trace amounts of Cre recombinase enzyme. Use of another AAV capsid with improved astrocyte tropism such as the PHP.A (Deverman et al., 2016) could further enhance the specificity of GEARBOCS.

GEARBOCS TagIN strategy enables both N and C-terminal tagging. However, an important caveat with introducing N-terminal tags is; it could lead to the gRNA-mediated KO of the gene if the donor integration does not happen. Similarly, GeneTRAP strategy targets donors with reporters close to the START codon and in the absence of donor integration the Cas9+ astrocytes could be KOs. Therefore, the neighboring unlabeled astrocytes, which express Cas9, should not be considered WT.

In the future, GEARBOCS method can be further developed and improved in several ways. GEARBOCS is a modular tool; thus, we can swap the promoters of gRNA and the Cre recombinase in a species- or cell type-specific manner to achieve broader applicability. For example, GEARBOCS can be modified to utilize smaller Cas9 homologs such as SaCas9 (Ran et al., 2015) under the gfaABC1D promoter instead of driving Cas9 expression via Cre recombinase. This would eliminate the need for using Cas9 transgenic mouse lines and permit the use of GEARBOCS in other species.

The efficiency of GEARBOCS-mediated KO, TagIN and GeneTRAP methods depend on many factors. The most important one is the effectiveness and specificity of the gRNA, which is heavily influenced by the accessibility of the targeted genomic locus. Off-target activity of gRNAs and imprecise insertion of donors pose significant challenges in CRISPR-mediated genome editing (Zhang et al., 2015). Therefore, careful validation of the gRNAs for the genes of interest are critical for experimental success. In this study, we used guides which were previously validated (Gao *et al.*, 2019; Horvath *et al.*, 2017). Future studies using GEARBOCS with newly developed guides should screen for off-target activity or precise insertion of tags at endogenous loci (Naeem et al., 2020).

In this study, we also investigated the expression, localization, and function of Vamp2 in astrocytes *in vivo* using GEARBOCS. Calcium-regulated vesicular exocytosis is a key feature of neuronal synapses and is mediated by the molecular machinery of the SNARE complex including Vamp2 (Salpietro *et al.*, 2019; Wang *et al.*, 2020). Several studies suggested that Vamp2, and other SNARE complex proteins, are involved in secretion of small neuroactive molecules and proteins from astrocytes (Verkhratsky et al., 2016; Araque et al., 2014; Harada et al., 2015). However, it has been difficult and controversial to provide evidence for Vamp2 expression and function in astrocytes (McMahon *et al.*, 1993; Slezak *et al.*, 2012; Yamamoto *et al.*, 2012). In this study, we used GEARBOCS to delineate the expression of Vamp2 in mouse cortical astrocytes *in vivo*. We were able to detect reporter expression under the control of Vamp2 endogenous promoter via GeneTRAP method and we found vesicle-like HA-tag staining within Vamp2-TagIN astrocytes. These data provide further evidence that Vamp2 is expressed by astrocytes *in vivo*.

GEARBOCS-mediated endogenous tagging and visualization of proteins such as Vamp2 circumvents the challenges and limitations of antibody-based immunolabeling of proteins in astrocytes *in vivo*. Thus, GEARBOCS facilitates the study of essentially any gene of interest in astrocytes, even in the absence of specific antibodies or conditional alleles. Moreover, this CRISPR tool allows for the determination of astrocyte-specific localization and distribution of proteins, even when the protein of interest, such as Vamp2, is a lot more abundant in another CNS cell type, such as neurons. In addition to these advantages, GEARBOCS can be used to replace the strategy of ectopic expression of tagged proteins which may alter their localization and function in astrocytes.

Astrocytes form an integral part of the synapse and control excitatory and inhibitory synaptogenesis through the secretion of synapse-modifying proteins (Chung *et al.*, 2015; Fossati et al., 2020; Lyon and Allen, 2021). Moreover, astrocytes have been proposed to participate in the regulation of neural circuits by secreting neuroactive small molecules such as D-serine and ATP that modify synaptic activity (Harada *et al.*, 2015; Haydon and Nedergaard, 2014). Because Vamp2 is well established to control synaptic neurotransmitter release in neurons (Salpietro *et al.*, 2019), as well as protein secretion in non-neuronal cell types (Mielnicka and Michaluk, 2021), we postulated that loss of Vamp2 will alter the regulation of excitatory and inhibitory synapse numbers locally. In agreement with this possibility, we found that loss of Vamp2 in astrocytes after the end of synaptogenic period (P21) significantly altered synapse numbers. Loss of astrocytic Vamp2 increased intracortical and thalamocortical excitatory synapse numbers within the territories of KO astrocytes, whereas the number of inhibitory synapses were reduced. These observations indicate that Vamp2-mediated secretion is required for maintaining the excitation/inhibition balance.

Astrocytes undergo local and global Ca^2+^-transients, albeit at much slower timescales than neurons. The astrocytic Ca^2+^-transients occur in response patterned neuronal activity and neuromodulators (Lim et al., 2021; Shigetomi et al., 2016). Manipulation of astrocytic calcium through opto- and chemo-genetic tools result in strong changes in synapse activity and animal behavior (Hirrlinger and Nimmerjahn, 2022). Vamp2 controls Ca^2+^-dependent exocytosis in neurons (Schoch et al., 2001; Takamori et al., 2006). Our results suggest that Vamp2 can also mediate astrocytic exocytosis of synapse-modifying molecules. Thus, our finding reveals a rich and unexplored aspect of astrocytic-secretion and provides the rationale for future studies to study Vamp2 function in astrocytes *in vivo*.

In summary, our results show that GEARBOCS is an effective and multifunctional gene-editing tool that offers a quick and efficient way to investigate astrocyte biology at the cellular and molecular levels in mice. The simple design of GEARBOCS make it a powerful and versatile ‘all-in-one’ CRISPR tool for both basic and translational research. Thus, future studies using GEARBOCS will likely provide new insights into the roles of astrocytes in the pathophysiology of neurodevelopmental and neurodegenerative diseases.

## Materials and Methods

**Table:** 

REAGENT or RESOURCES	SOURCE	IDENTIFIER

**Antibodies**		

HA	Roche	Cat# 11867423001, RRID:AB_390918
GFP	Aves Labs	Cat# GFP-1020, RRID:AB_10000240
RFP	Rockland	Cat# 600-401-379, RRID:AB_2209751
Sox9	Millipore	Cat# AB5535, RRID:AB_2239761
Vamp2	Proteintech	Cat# 10135-1-AP, RRID:AB_2256918
NeuN	Millipore	Cat# MAB377, RRID:AB_2298772
Olig2	Millipore	Cat# MABN50, RRID:AB_10807410
mCherry	Aves Labs	Cat #MCHERRY-0020
Vamp2	Synaptic Systems	Cat# 104 403, RRID:AB_2864782
VGAT	Synaptic Systems	Cat# 131 004, RRID:AB_887873
PSD95	Thermo Fisher Scientific	Cat# 51-6900, RRID:AB_2533914
VGLUT1	Millipore	Cat# AB5905, RRID:AB_2301751
VGLUT2	Synaptic Systems	Cat# 135 404, RRID:AB_887884
Gephyrin	Synaptic Systems	Cat# 147 011, RRID:AB_887717
Rabbit Alexa Fluor^™^ 488	Invitrogen	Cat#A-11034
Guinea pig Alexa Fluor^™^ 647	Invitrogen	Cat#A-21450
Chicken Alexa Fluor^™^ 488	Invitrogen	Cat#A-11039
Chicken Alexa Fluor^™^ 594	Invitrogen	Cat#A-11042
Rabbit Alexa Fluor^™^ 594	Invitrogen	Cat#A-11037
Rat Alexa Fluor^™^ 594	Invitrogen	Cat#A-11007
Mouse Alexa Fluor^™^ 594	Invitrogen	Cat#A-21125
Mouse Alexa Fluor^™^ 568	Invitrogen	Cat#A-21134
Mouse Alexa Fluor^™^ 488	Invitrogen	Cat#A-21121
Rat Alexa Fluor^™^ 568	Invitrogen	Cat#A-11077
Mouse Alexa Fluor^™^ 647	Invitrogen	Cat#A-21240

**Bacterial and virus strains**		

One Shot^™^ Stbl3^™^ Chemically Competent E. coli	Invitrogen	Cat# C737303
AAV-GEARBOCS	This Study	NA
AAV-GEARBOCS-*Gfap*-KO	This Study	NA
AAV-GEARBOCS-*Gfap*-TagIN-mCherry	This Study	NA
AAV-GEARBOCS-*Gfap*-TagIN-HA	This Study	NA
AAV-GEARBOCS-*Gfap*-GeneTRAP	This Study	NA
AAV-GEARBOCS-*Vamp2*-GeneTRAP	This Study	NA
AAV-GEARBOCS-*Vamp2*-TagIN-HA	This Study	NA
AAV-gfaABC1D-mCherry-CAAX	This Study	NA

**Chemicals, Peptides, and Recombinant Proteins**		

PEI MAX^®^	Polysciences	Cat#24765
Optiprep	Sigma	Cat# D1556
Pen/Strep	GIBCO	Cat# 15140
Sodium Pyruvate	GIBCO	Cat# 11360-070
L-Glutamine	GIBCO	Cat# 25030-081
DMEM	GIBCO	Cat# 11960044
DPBS	GIBCO	Cat# 14190144
Benzonase	Novagen	Cat#70664
Goat Serum	GIBCO	Cat#16210064
Triton^™^ X-100 Surfact-Amps^™^ Detergent Solution	Thermo Scientific	Cat#28314
Fetal Bovine Serum	Sigma	Cat#F4135

**Critical Commercial Assays**		

Endo-Free Maxi Prep Kit	QIAGEN	Cat# 12362
QIAprep Spin Miniprep Kit	QIAGEN	Cat# 27106
QIAquick Gel Extraction Kit	QIAGEN	Cat# 28704
Vivaspin^™^ ultrafiltration spin columns	Cytiva	Cat# 28932363
Zero Blunt^™^ TOPO^™^ PCR Cloning Kit	Invitrogen	Cat# 450031
In-Fusion^®^ Snap Assembly Master Mix	Takara	Cat# 638948
Fast SYBR^™^ Green Master Mix	Applied Biosystems^™^	Cat#4385612
Phusion^®^ High-Fidelity PCR Kit	NEB	Cat# E0553L

**Experimental models: cell lines**		

HEK293T	ATCC	CRL-11268

**Experimental models: organisms/strains**		

B6J.129(B6N)-Gt(ROSA)26Sortm1(CAG-cas9*,-EGFP)Fezh/J	Jackson Laboratory	Cat#026175
B6.129-Igs2tm1(CAG-cas9*)Mmw/J	Jackson Laboratory	Cat#027632

**Oligonucleotides**		

*Gfap* gRNA1	This Study	See Methods
*Gfap* gRNA2	This Study	See Methods
*Vamp2* gRNA	This Study	See Methods

**Software and algorithms**		

GraphPad Prism 9.4.1	GraphPAD	https://www.graphpad.com/scientific-software/prism/
ImageJ	NIH	https://imagej.nih.gov/ij/
Syn_Bot	Eroglu Lab, Duke University	https://github.com/Eroglu-Lab/Syn_Bot
Imaris 9.9.0	BitPlane	https://imaris.oxinst.com/packages
JMP^®^ Pro 17	SAS	https://www.jmp.com
R	The R Foundation	https://www.r-project.org/
ilastik	Kreshuk Lab, European Molecular Biology Laboratory	https://www.ilastik.org/index.html
CRISPick	Broad Institute	https://portals.broadinstitute.org/gppx/crispick/public

**Recombinant DNA**		

pZac2.1-GfaABClD-Lck-GCaMP6f	Addgene	Cat#52924
pGEARBOCS	This Study	Addgene # 196495
pGEARBOCS-*Gfap*-KO	This Study	Addgene # 196489
pGEARBOCS-*Gfap*-TagIN-mCherry	This Study	Addgene # 196490
pGEARBOCS-*Gfap*-TagIN-HA	This Study	Addgene # 196491
pGEARBOCS-*Gfap*-GeneTRAP	This Study	Addgene # 196492
pGEARBOCS-Vamp2-GeneTRAP	This Study	Addgene # 196493
pGEARBOCS-Vamp2-TagIN-HA	This Study	Addgene # 196494
pgfaABC1D-mCherry-CAAX	This Study	Addgene # 196488

### Resource availability

#### Lead contact

Further information and requests for resources and reagents should be directed to the lead contact, Cagla Eroglu (cagla.eroglu@duke.edu).

### Experimental model and subject details

#### Animals

All mice experiments were carried out under a protocol approved by the Duke University Institutional Animal Care and Use Committee (IACUC) in accordance with US National Institutes of Health guidelines. All mice were housed in the Duke Division of Laboratory Animal Resources (DLAR) facility and follow typical day/night conditions of 12-hours cycles. Both B6J.129(B6N)-Gt (ROSA)26Sortm1(CAG-cas9*,EGFP)Fezh/J (JAX stock #026175) and B6.129-Igs2tm1(CAG-cas9*)Mmw/J (JAX stock #027632) mice were purchased from Jackson laboratory. AAV retro-orbital injections were performed at P21, and brains collected at P42. Sex-matched littermate pairs (both male and female) were randomly assigned to experimental groups for all experiments.

#### Plasmids and CRISPR guides

To generate GEARBOCS, Cre expression cassette was cloned first into the pZac2.1-GfaABC1D-Lck-GCaMP6f (A gift from Dr. Baljit Khakh; Addgene plasmid #52924) by replacing Lck-GCaMP6f. U6 expression cassette along with the donor insertion sites (DIS) were synthesized as gBlocks (IDT) and cloned upstream of gfaABC1D promoter to generate GEARBOCS. pAAV-gfaABC1D-mCherry-CAAX was generated by cloning mCherry-CAAX into pZac2.1-GfaABC1D-Lck-GCaMP6f by replacing Lck-GCaMP6f. All the gRNAs used in this study were cloned into the Sap1 site of GEARBOCS and the donors were cloned between the Sal1 and BamH1 site in the DIS. To make the GEARBOCS donors, mCherry donors were PCR amplified and HA donors were oligo annealed with overhangs to clone into the DIS. All generated plasmids were confirmed by Sanger sequencing protocol. The sgRNA sequences used in this study were as follows: Gfap: gRNA1: 5’-GAGGCCCCTGACCACCGTCTCGG-3’ gRNA2: 5’-TATCTAAGGGAGAGCTGGCAGGG-3’ and Vamp2: 5’- GGCCGGGGCGGCAGGCGGGACGG-3’.

#### AAV production and purification

Purified AAVs were produced as previously described (Uezu et al., 2016). Briefly, HEK293T cells grown on 150mm dishes were transfected with GEARBOCS plasmid, helper plasmid pAd-DeltaF6 and the serotype plasmid AAV PHP.eB. After three days, cell lysates were prepared with 15mM NaCl, 5mM Tris-HCl, pH 8.5 followed by three repeats of freeze-thaw cycles. The cell lysates were centrifuged for 30min at 4000rpm to collect the supernatant. Benzonase-treated supernatant (50U/ml, 30 min at 37°C) was added to an Optiprep density gradient (15%, 25%, 40% and 60%) for ultracentrifugation at 60,000 rpm for 1.5hr using a Beckman Ti-70 rotor. AAV fraction was collected from the gradient and concentrated along with the multiple washes with DPBS in a 100 kDa filtration unit. AAV titers were quantified by qPCR based on SYBR green technology using primer pair targeting AAV2 ITR (Aurnhammer et al., 2012).

#### AAV injection and tissue preparation

P21 Cas9 or Cas9-EGFP mice placed in a stereotaxic frame were anesthetized through inhalation of 1.5% isoflurane gas. 10μl of purified AAVs (titer of ~1 × 10^12^ GC/ml) was intravenously injected into the retro-orbital sinus. After 3 weeks at P42, mice were anesthetized with 200 mg/kg Tribromoethanol (Avertin) and transcardially perfused with ice-cold TBS/Heparin and 4% paraformaldehyde (PFA) at room temperature (RT). Harvested brains were post-fixed overnight in 4% PFA, cryoprotected in 30% sucrose and the brain blocks were prepared with O.C.T. (TissueTek) to store at −80°C. 25μm thick brain sections were obtained through cryo-sectioning using a Leica CM3050S (Leica, Germany) vibratome and stored in a mixture of TBS and glycerol at −20°C for further free-float antibody staining procedures.

#### HEK293T cell culture

For AAV production, HEK293T (ATCC CRL-11268) cells were cultured in DMEM high glucose medium (Thermo Fisher #11960044) supplemented with 2mM L-Glutamine, 10% fetal bovine serum, 100U/ml Penicillin-Streptomycin, and 1 mM sodium pyruvate. Cells were incubated at 37°C in 5% CO2 and passaged by trypsin/EDTA digestion upon reaching ~95% confluency. Cells were transfected with PEI Max (Polysciences) when reaching 60–80% confluency.

#### Immunohistochemistry

For immunohistochemistry, frozen tissue sections were washed three times in 0.2% TBST (0.2% Triton X-100 in 1x TBS). The sections were blocked and permeabilized with a blocking buffer (5% normal goat serum in 0.2% TBST) for 1hr at RT followed by the overnight incubation with the primary antibodies at 4°C. Primary antibodies were diluted in blocking buffer. After primary incubation, sections were washed in 0.2 % TBST and incubated with fluorescent secondary antibodies diluted in blocking buffer for 2–3 hours at RT. Tissue sections were washed in TBST and mounted on glass slides with Vectashield with DAPI (Vector Laboratories, CA). Primary antibodies used for immunohistochemistry were listed as following: Rat anti-HA (Roche/Sigma #11867423001), Chicken anti-GFP (Aves Labs #GFP-1020), Rabbit anti-RFP (Rockland #600-401-379), Rabbit anti-Sox9 (Millipore #AB5535), Rabbit anti-Vamp2 (Proteintech # 10135-1-AP), Mouse anti-NeuN (Millipore # MAB377), Mouse anti-Olig2 (Millipore #MABN50), Chicken anti-mCherry (Aves Labs #MCHERRY-0020), Mouse anti-Vamp2 (Synaptic Systems # 104211), Guinea pig anti-VGAT (Synaptic Systems #131004), Rabbit anti-PSD95 (Thermo Fisher Scientific #51-6900), Guinea pig anti-VGLUT1 (Millipore # AB5905), Guinea pig anti-VGLUT2 (Synaptic Systems #135 404) and Mouse anti-gephyrin (Synaptic Systems #147-011). Goat Alexa Fluor Secondary antibodies (Invitrogen) used for immunohistochemistry were listed as following: Rabbit 488 (#A-11034), Guinea pig 647(#A-21450), Chicken 488(#A-11039), Chicken 594 (#A-11042), Rabbit 594 (#A-11037), Rat 594(#A-11007), Mouse 594(#A-21125), Mouse 568 (#A-21134), Mouse 488 (#A-21121), Rat 568 (#A-11077) and Mouse 647 (#A-21240). To avoid excessive background staining, isotype specific secondary antibodies were used for primary antibodies produced in mice.

#### Synaptic Puncta Colocalization Analysis

Confocal images of pre- and postsynaptic puncta were prepared for analysis using ImageJ (https://imagej.nih.gov/ij/) to convert the raw images into RGB type images with the presynaptic marker (VGlut1/VGlut2 or VGAT) in the red channel, the postsynaptic marker in the green channel (PSD95 or Gephyrin), and the astrocyte marker (mCherry) in the blue channel. Images were then Z-projected to make one projection for every 3 images, representing 1 um of depth in our imaging setup. Several of these Z-projections were then used to train a random forest model using the ilastik software for thresholding images of each of the synaptic markers. To restrict the analysis to the territory of a transduced astrocyte, each image was manually cropped. An ImageJ macro for counting colocalized synaptic puncta, SynBot [https://github.com/Eroglu-Lab/Syn_Bot], was then used to count the number of colocalized synaptic puncta in each of the cropped images using the corresponding ilastik models to threshold each image channel. The same ilastik models and parameters were used to analyze the different groups within each experiment. Colocalized synaptic puncta counts were then normalized to the area of the corresponding cell territory. Colocalized synaptic puncta density was then averaged for the images from each animal used in the experiment.

#### Neuropil infiltration volume analysis

Astrocyte morphology was analyzed by calculating the volume of peri-synaptic astrocyte processes present in the surrounding neuropil. Immunostained astrocytes overexpressing mCherry-CAAX were imaged by confocal microscopy. High magnification (63x plus 2x optical zoom) 15um Z-stack micrographs of individual astrocytes were acquired using the Olympus Fluoview FV3000 confocal microscope by imaging the middle 1/3 of the astrocyte containing the cell soma and surrounding arbor. The images were reconstructed on Imaris Bitplane 9.9.0 software for 3D reconstructions. For every astrocyte analyzed, three 9.45μm × 9.45μm × 5.25μm regions of interest (ROIs, 75 pixels × 75 pixels × 15 pixels) devoid of the soma and large branches were reconstructed using the surface tool in Imaris, similar to (Stogsdill *et al.*, 2017). Astrocyte NIVs were statistically analyzed using Nested t-test.

#### Statistical Analysis

For data collection, brains from the healthy mice in each experimental group were collected and processed. All data are represented as mean ± standard error of the mean and P < 0.05 were considered to indicate statistical significance. Exact P-values are noted in the figure/figure legends for each experiment. Statistical analyses were performed with JMP Genomics Pro 17.0 software, and the graphs were generated with GraphPad Prism version 9 (GraphPad Software). Statistical analyses of the quantified data were performed using either Nested analysis of variance (ANOVA) or Nested t-test. Sample size for each experiment and statistical method of analysis are indicated in the figure legend for each experiment.
