# Supplementary material for: GEARBOCS: An Adeno Associated Virus Tool for In Vivo Gene Editing in Astrocytes
Source: bioRxiv. 2024 Sep 14:2023.01.17.524433. Originally published 2023 Jan 19. Preprint. [Version 3] doi: 10.1101/2023.01.17.524433 (PMC9884502; doi:10.1101/2023.01.17.524433)
Supplement: Supplementary file 1 [file media-1.pdf]

Resources table

**REAGENT or RESOURCE**	SOURCE	IDENTIFIER
**Antibodies**		
Rat-anti-HA	Roche	Cat# 11867423001, RRID:AB_390918
Chicken-anti-GFP	Aves Labs	Cat# GFP-1020, RRID:AB_10000240
Rabbit-anti-RFP	Rockland	Cat# 600-401-379, RRID:AB_2209751
Rabbit-anti-Sox9	Millipore	Cat# AB5535, RRID:AB_2239761
Rabbit-anti-Vamp2	Proteintech	Cat# 10135-1-AP, RRID:AB_2256918
Mouse-anti-NeuN	Millipore	Cat# MAB377, RRID:AB_2298772
Mouse-anti-Olig2	Millipore	Cat# MABN50, RRID:AB_10807410
Chicken-anti-mCherry	Aves Labs	Cat #MCHERRY-0020
Mouse-anti-Vamp2	Synaptic Systems	Cat# 104 403, RRID:AB_2864782
Mouse-anti-VGAT	Synaptic Systems	Cat# 131 004, RRID:AB_887873
Rabbit-anti-PSD95	Thermo Fisher Scientific	Cat# 51-6900, RRID:AB_2533914
Guinea Pig-anti-VGLUT1	Millipore	Cat# AB5905, RRID:AB_2301751
Guinea Pig-anti-VGLUT2	Synaptic Systems	Cat# 135 404, RRID:AB_887884
Mouse-anti-Gephyrin	Synaptic Systems	Cat# 147 011, RRID:AB_887717
Rabbit Alexa Fluor™ 488	Invitrogen	Cat# A-11034 RRID: AB_2576217
Guinea pig Alexa Fluor™ 647	Invitrogen	Cat# A-21450 RRID: AB_2535867
Chicken Alexa Fluor™ 488	Invitrogen	Cat# A-11039 RRID: AB_2534096
Chicken Alexa Fluor™ 594	Invitrogen	Cat# A-11042 RRID: AB_2534099
Rabbit Alexa Fluor™ 594	Invitrogen	Cat# A-11037 RRID: AB_2534095
Rat Alexa Fluor™ 594	Invitrogen	Cat# A-11007 RRID: AB_10561522
Mouse Alexa Fluor™ 594	Invitrogen	Cat# A-21125 RRID: AB_2535767

Mouse Alexa Fluor™ 568	Invitrogen	Cat# A-21134 RRID: AB_2535773
Mouse Alexa Fluor™ 488	Invitrogen	Cat# A-21121 RRID: AB_2535764
Rat Alexa Fluor™ 568	Invitrogen	Cat# A-11077 RRID: AB_2534121
Mouse Alexa Fluor™ 647	Invitrogen	Cat# A-21240 RRID: AB_2535809
**Bacterial and virus strains**		
One Shot™ Stbl3™ Chemically Competent E. coli	Invitrogen	Cat# C737303
AAV-GEARBOCS-v0	This Study	NA
AAV-GEARBOCS (with 4xmiRT)	This Study	NA
AAV-GEARBOCS-Sparcl1-KO	This Study	NA
AAV-GEARBOCS-Sparcl1-TagIn-C-mCherry	This Study	NA
AAV-GEARBOCS-Sparcl1-TagIn-N-mCherry	This Study	NA
AAV-GEARBOCS-Sparcl1-TagIn-N-HA	This Study	NA
AAV-GEARBOCS-Sparcl1_GeneTRAP	This Study	NA
AAV-GEARBOCS-*Vamp2*-GeneTRAP	This Study	NA
AAV-GEARBOCS-v0-*Vamp2*-TagIN-HA	This Study	NA
AAV-GfaABC1D-mCherry-CAAX	This Study	NA
**Chemicals, Peptides, and Recombinant Proteins**		
PEI MAX®	Polysciences	Cat#24765
Optiprep	Sigma	Cat# D1556
Pen/Strep	GIBCO	Cat# 15140
Sodium Pyruvate	GIBCO	Cat# 11360-070
L-Glutamine	GIBCO	Cat# 25030-081
DMEM	GIBCO	Cat# 11960044

DPBS	GIBCO	Cat# 14190144
Benzonase	Novagen	Cat#70664
Goat Serum	GIBCO	Cat#16210064
Triton™ X-100 Surfact-Amps™ Detergent Solution	Thermo Scientific	Cat#28314
Fetal Bovine Serum	Sigma	Cat#F4135
2,2,2 tribromoethanol (Avertin)	Sigma	Cat# T48402-25G
2-methyl-2-butanol	Sigma	Cat# 152463-250mL
Opti-MEM	GIBCO	Cat# 31985070
X-tremeGENE HP DNA Transfection Reagent	Sigma-Aldrich	Cat# 6366244001
PFA 16%	Electron Microscopy Sciences	Cat# 15710
Tissue-Tek O.C.T. Compound	Sakura Finetek	Cat# 4583
Heparin	Sigma	Cat# H3149-10KU
**Critical Commercial Assays**		
Endo-Free Maxi Prep Kit	QIAGEN	Cat# 12362
QIAprep Spin Miniprep Kit	QIAGEN	Cat# 27106
QIAquick Gel Extraction Kit	QIAGEN	Cat# 28704
Vivaspin™ ultrafiltration spin columns	Cytiva	Cat# 28932363
Zero Blunt™ TOPO™ PCR Cloning Kit	Invitrogen	Cat# 450031
In-Fusion® Snap Assembly Master Mix	Takara	Cat# 638948
Fast SYBR™ Green Master Mix	Applied Biosystems™	Cat#4385612
Phusion® High-Fidelity PCR Kit	NEB	Cat# E0553L
DNeasy Blood & Tissue Kit	QIAGEN	Cat# 69504

**Experimental models: cell lines**		
HEK293T	ATCC	CRL-11268
NIH3T3/Cas9 Cell Line	Calibre Scientific	CBIO-AKR-5104
**Experimental models: organisms/strains**		
B6J.129(B6N)-Gt (ROSA)26Sortm1(CAG-cas9*,-EGFP)Fezh/J	Jackson Laboratory	Cat#026175
B6.129-Igs2tm1(CAG-cas9*)Mmw/J	Jackson Laboratory	Cat#027632
**Oligonucleotides**		
Sparcl1 gRNA	This Study	See Methods
Vamp2 gRNA	This Study	See Methods
**Equipment**		
Beckman Ti70 rotor		
Beckman preparative ultracentrifuge		
U-100 Insulin syringes	BD	Cat# 324702
**Software and algorithms**		
GraphPad Prism 9.4.1	GraphPAD	https://www.graphpad.com/scientific-software/prism/ RRID: SCR_002798
ImageJ	NIH	https://imagej.nih.gov/ij/ RRID: SCR_003070
Puncta Analyzer (version 2.0)	Ippolito and Eroglu, 2010	https://github.com/toddstavish/puncta-analyzer RRID: SCR_025425
Imaris 9.9.0	BitPlane	https://imaris.oxinst.com/packages RRID: SCR_007370
JMP® Pro 17	SAS	https://www.jmp.com RRID: SCR_022199
R	The R Foundation	https://www.r-project.org/ RRID: SCR_001905

CRISPick	Broad Institute	https://portals.broadinstitute.org/gppx/crispick/public
**Recombinant DNA**		
AAV-PHP.eB_capsid	Addgene	RRID:Addgene_103005
pAD-ΔF6	Addgene	RRID:Addgene_112867
pZac2.1-GfaABC1D-Lck-GCaMP6f	Addgene	Cat#52924
pGEARBOCS-v0	This Study	Addgene #: 196495
pGEARBOCS (with 4xmiRT)	This Study	Addgene #: 218181
pGEARBOCS-Sparcl1-KO	This Study	Addgene #: 218182
pGEARBOCS-Sparcl1-TagIn-C-mCherry	This Study	Addgene #: 218183
pGEARBOCS-Sparcl1-TagIn-N-mCherry	This Study	Addgene #: 218186
pGEARBOCS-Sparcl1-TagIn-N-HA	This Study	Addgene #: 218185
pGEARBOCS-Sparcl1_GeneTRAP	This Study	Addgene #: 218184
pGEARBOCS-*Vamp2*-GeneTRAP	This Study	Addgene #: 218187
pGEARBOCS-v0-*Vamp2*-TagIN-HA	This Study	Addgene #: 196494
pGfaABC1D-mCherry-CAAX	This Study	Addgene #: 218189
**Other**		
Centrifugation tubes for freeze-thawing: with centristar cap	Corning	Cat# 430828
Ultracentrifuge sealing tubes, optiseal	Beckman	Cat# 361625
